# A prospective study investigating gross motor function of children with cerebral palsy and GMFCS level II after long-term Botulinum toxin type A use

**DOI:** 10.1186/s12887-019-1906-8

**Published:** 2020-01-06

**Authors:** Jane Valentine, Sue-Anne Davidson, Natasha Bear, Eve Blair, Lisa Paterson, Roslyn Ward, David Forbes, Catherine Elliott

**Affiliations:** 1 School of Medicine, University of Western Australia, 35 Stirling Highway, Crawley, Western Australia 6009 Australia; 2 Department of Paediatric Rehabilitation, Perth Children’s Hospital, Nedlands, Western Australia Australia; 3 Department of Child Research, Child Adolescent Health Service, Nedlands, Western Australia Australia; 4 Telethon Kids Institute, Perth, Western Australia Australia; 5 School of Occupational Therapy, Social Work and Speech Pathology, Curtin University, Bentley, Western Australia Australia

## Abstract

**Background:**

The aim of this study is to contribute to the knowledge base on the long-term outcomes of evidence-based medical interventions used to improve gross motor function in children and adolescents with Cerebral Palsy.

**Method:**

Prospective cohort study of children with Cerebral Palsy in the birth years 2000–2009 attending a tertiary level service for children with Cerebral Palsy who’s first recorded Gross Motor Function Classification System level was II.

**Results:**

A total of 40 children were eligible for the study, of whom 28 (72.7%) enrolled. The Botulinum toxin A treatment for this cohort, (median and interquartile ranges) were: total number of lower limb Botulinum toxin A injections 11 (6.7, 5.5); total dose of Botulinum Toxin A per lower limb treatment 6.95 u/kg (4.5, 11); and dose of Botulinum Toxin u/kg/muscle 2.95 (2.2, 4). For all 28 subjects there was a median of 15 (8.5 to 22) Gross Motor Function Classification System level recordings: six of the 28 children (21.4%) improved from level II to level I, the remaining 22 children remained stable at level II (78.6%). In this highly treated population, the average 66 item Gross Motor Function Measure score for the 22 children in level II was 72.55, which is consistent with the mean of 68.5 reported in the original Ontario cohort.

**Conclusion:**

This cohort study has confirmed that children with Cerebral Palsy, Gross Motor Function level II treated at a young age with repeated doses of Botulinum Toxin A within an integrated comprehensive service, maintain or improve their functional motor level at a later age.

## Background

Cerebral Palsy (CP), the most common motor disorder of childhood, was described by Rosenbaum et al. in 2007 as a ‘group of permanent disorders of the development of movement and posture that are attributed to non-progressive disturbances that occurred in the developing fetal or infant brain’ [1, 2]. The Australian Cerebral Palsy Register has recorded the prevalence of CP as 2.1 per 1000 live births [3]. Perth Children’s Hospital (PCH), Western Australia (WA) (population 2.4 million) is the state centre for the management of motor disorders in children and adolescents with CP. In 2003 following new funding from the West Australian government an evidence-based clinical program for children with CP in WA was established and is known as the Cerebral Palsy Mobility Service (CPMS). As part of the funding a database, the Paediatric Rehabilitation Information System (PRIS), was established, with retrospective data entry for children with CP to 1995 [4]. In a recent retrospective audit, we confirmed that the CPMS manages the state-wide population of children and adolescents with CP in WA and provides accurate tracking of interventions [4].

Motor development, pain and integration into community life are primary concerns of parents of children with CP [5] and interventions including Botulinum toxin type A (BoNTA) and orthopaedic surgery are designed to improve motor function to allow participation, treat pain and prevent secondary impairments. BoNTA is an evidence based management for hypertonia in children with CP [2, 6, 7]. BoNTA has been used to manage hypertonia in children with CP since 1993 [8]. BoNTA has a high safety profile [9–11] and the short-term outcomes of BoNTA are well documented [12–20]. Molenaers et al., concluded that when injected according to an integrated approach and started at a young age, BoNTA has the potential to improve overall function of children with CP [21]. However as documented by Kahraman in their systematic review and others there is little evidence on the outcome of repeated BoNTA injections over time [22–24] and the long-term effect of BoNTA on muscle size and morphology in children with CP remains under investigation [25, 26].

The evidence base for interventions that are proven effective in children with CP is limited with the majority of interventions only having evidence for short-term gains [7]. Two recent Delphi surveys of consumers, researchers and clinicians have identified the need to provide evidence of longer-term outcomes of interventions for children with CP [27, 28].

## Methods

The aim of this study is to contribute to the knowledge base on the long-term outcomes of evidence-based medical interventions used to improve motor function in children and adolescents with CP. In this cohort study we will compare the observed gross motor function profiles of children with CP whose first recorded Gross Motor Function Classification System (GMFCS) level was level II and who are currently aged between 8 and 16 years and enrolled in the CPMS, with their predicted average 66 item Gross Motor Function Measure (GMFM-66) score on the Ontario Motor Growth Curves [29] for their current GMFCS level. We will also measure the pain and participation levels of these children. The primary question is: ‘Do children treated at a young age with repeated doses of BoNTA within an integrated comprehensive service, maintain their functional motor gains at a later age?’ The secondary question is: ‘What are the comorbidities, pain and participation profiles of these children?’

This prospective cohort study includes children with CP whose first recorded GMFCS level was level II, who are in the birth cohort 2000–2009 inclusive (aged 8–16 years at time of assessment) and currently enrolled in the CPMS. Exclusion criteria included a lack of GMFCS level recorded at time point 1, BoNTA treatment external to our CPMS service, history of selective dorsal root rhizotomy, declining to participate or inability to comply with assessments. Data concerning enrolled children was obtained at two time points: time point 1, the time of entry of the child into the CPMS for treatment; and time point 2, is at the date of motor assessments and questionnaires.

Data for time point 1 is data taken from the CPMS database records and includes topographical classification (hemiplegia, diplegia etc.), comorbidities and GMFCS level. Data for time point 2 includes the functional motor assessments GMFCS and GMFM-66. The GMFM-66 is a valid and reliable measure [30], we used the Gross Motor Ability Estimator (GMAE-2) computer program to estimate a total GMFM-66 score [30]. To interpret the GMFM-66 scores, we compared the score to the predicted GMFM-66 score for the GMFCS level on the ‘Percentiles by Age’, Ontario Motor Growth Curves [31].

The Brief Pain Inventory – Short Form [32] was used to record pain history. This questionnaire comprises two parts: the first part contains eight items regarding pain location, pain severity, analgesics used and pain relief; the second part asks the individual about pain interference with activities in daily life. The Participation and Environment Measure for Children and Youth (PEM-CY) short form was used to measure activity and participation [33]; this is a parent-report instrument that examines participation and environment factors that affect the participation of children across three settings: home, school and community. Parents are asked to rate their child’s involvement in 25 activities across the three settings. A questionnaire concerning medication use was completed and anthropometric measures to calculate body mass index (BMI) z scores were also taken. All assessments were done by a qualified physiotherapist (author LP) while the patient was attending an outpatient clinic.

At time point 2, data was also extracted from PRIS concerning the date of birth of the child and the date, type and GMFCS level for each intervention. In view of our focus on GMFCS in this report, the details for BoNTA intervention data was limited to the lower limb use. BoNTA data extracted included muscle treatment site(s), total lower limb dose of BoNTA (u/kg), BoNTA dose per muscle (u/kg/muscle), and indications for use of BoNTA. Our service has only ever used Onabotulinum toxin (Allergan) as our BoNTA treatment. Treatment sites are recorded as distal if involving muscles that insert below the knee joint, proximal if muscles insert above the knee (including psoas) and multilevel if both distal and proximal muscles were treated. Indications for use of BoNTA were recorded as ‘improve function’, ‘manage symptoms’ (including pain and splint tolerance) and ‘care and comfort’. It is possible to have multiple indications for each treatment. The data fields concerning indications for use were only introduced into the database in 2013 so data for this field is incomplete. In view of our focus on GMFCS, in this report only the details for lower limb orthopaedic surgery are provided and indications for surgery are coded as hip only, gait only, function only or a combination of these.

### Data analysis

Age was calculated in months and then converted to years from the date of birth. The GMFCS level was classified initially according to Palisano (1997) and from 2007 onwards according to the GMFCS Extended and Revised version [29, 34]. Although the GMFCS level was assessed at each clinic visit, it is updated on PRIS only if the GMFCS level changes. To assess GMFCS level stability, the first and last GMFCS level recorded were compared. Children and adolescents who no longer need treatment by the CPMS are discharged from the service but are eligible for re-referral from the community if required. The reasons for discharge include stable function with no further treatment considered likely to be needed, patient deceased or, for a very small number, relocation of the family.

Continuous variables are reported as means and standard deviations or medians and interquartile ranges (when distributions were skewed). Categorical variables are reported as frequencies and percentages. Relationships between categorical variables (BoNTA use in multilevel muscles vs topography, pain and BMI) were compared using chi square test. PEMCY and topography, GMFCS, pain and BMI (categories) was compared using the Mann Whitney U test. PEMCY and GMFM was analysed using linear regression and Pearsons correlation coefficients. All data was analysed using Stata 14.1 (StataCorp, College Station, TX). Statistical significance was considered *p* < 0.05.

## Results

**Fig. 1:**
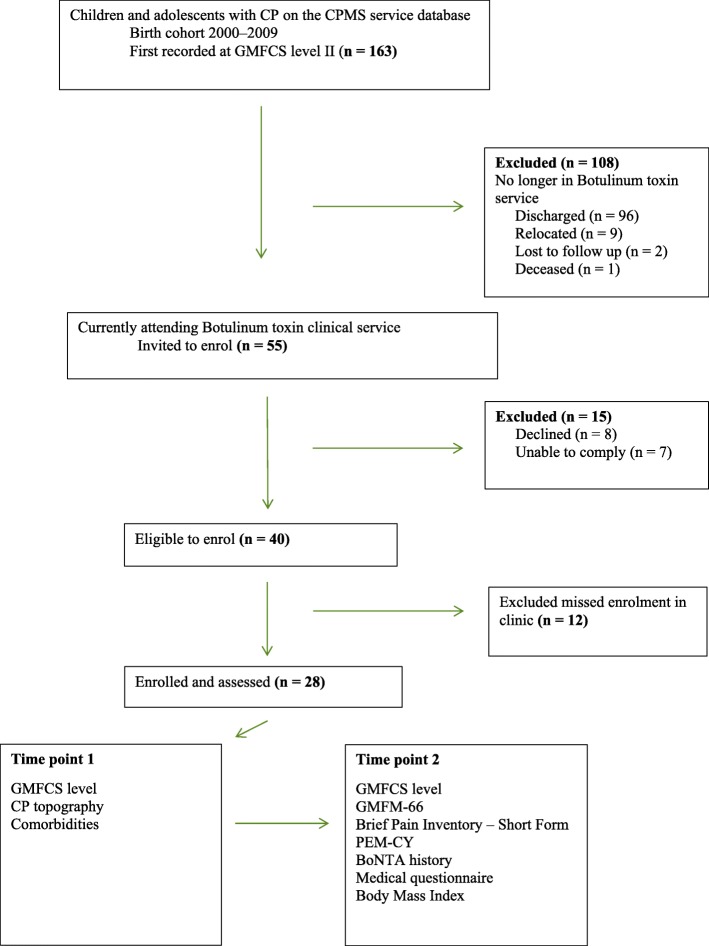
Flow diagram of enrolment

There were 766 children aged between 8 and 16 years at the time of assessment. At time point 1, 163 of these children (21.3%) were recorded at GFMCS level II. Figure 1 outlines the patient flow through the clinical service. There were 55 individuals potentially eligible for enrolment in the study; of these, 15 (27.3%) were excluded as they either declined (*n* = 8) or they were unable to comply (*n* = 7) due to comorbidities, including autism and intellectual disability. A total of 40 children were eligible, of whom 28 (72.7%) were enrolled and assessed. These 28 children represent a sample of convenience of the total 40 children who could be assessed as the study was conducted over a limited time period in a busy clinical service.

Of the 108 children no longer in the service, the majority (96 children) were discharged as their motor function was stable and it was considered they were unlikely to require future BoNTA treatment. Of these 108 children, only 65 (60%) had ever received BoNTA. Of these 65 children, the median (IQR [interquartile range]) age at last dose of BoNTA was 9 (6, 11) years with a median (IQR) of 5 (2, 12) lower limb BoNTA treatments. For these 108 children, 24 (22%) had improved to become GMFCS level I, 72 (67%) were stable at GMFCS level II, and 12 (11%) deteriorated – 10 to GMFCS level III and two to GMFCS level IV.

The median (IQR) age of the 108 excluded children was 13.3 (11.7, 15.6) years compared with 11.5 (10, 12.7) years of the 55 potentially eligible children. 54 of the 55 (98%) of the eligible children received BoNTA. Table 1 compares CP topography, comorbidity rates and BoNTA use between the 55 enrolled and non-enrolled potentially eligible children. These two groups were similar in topography, age and comorbidities rate but the median number of BoNTA treatments was lower in the non-enrolled group.

**Table 1:** Enrolled and non-enrolled potentially eligible patients

Group N	CP Type N (%)	Last GMFCS N (%)	Comorbidity = Yes N (%)	Total number lower limb BoNTA received by an individual Median (IQR)	Indication^a^ N	Age (yrs) first BoNTA Median (IQR)	Age (yrs) last BoNTA Median (IQR)	N (%) children undergoing surgery N by indication Age (yrs) first surgery Median (IQR)
Non-enrolled 27	Hemiplegia 9 (34)	Stable 17 (63)	12 (44.4)	7 (5.5, 14.5)	Improve function 15	4 (2, 6)	10 (8, 11)	8 (30) Total 5 Gait only 1 Hips only 2 Hip and Gait 7.5 (4, 11)
	Diplegia 16 (59)	Improved 7 (26)			Manage symptoms 3			
	Quadriplegia 2 (7)	Deteriorated 3 (11)			Care and comfort 2			
Enrolled 28	Hemiplegia 13 (46)	Stable 22 (78)	15 (54)	11 (6.71, 5.5)	Improve function 23	3.5 (2, 6)	10 (8.5, 11)	4 (14) Gait only 9 (7, 11)
	Diplegia 15 (54)	Improved 6 (22)			Manage symptoms 1			
		Deteriorated 0 (0)						

^**a**^The data fields concerning these indications for use were introduced into the database only in 2013 so data for this field is incomplete

Table 2 details CP topography predominate motor type, comorbidities, functional levels, BoNTA dosing, muscle injection level distribution and GMFM scores for the 28 enrolled children. Their median (IQR) age was 10.9 (10, 11.8) years. Diplegia topography was observed in 15 (53.6%) children and hemiplegic in 13 (46.4%) children. Comorbidity rates were high with 15 (54%) of the group reported to have comorbidities that included intellectual disability, epilepsy and autism. The mean BMI z score was 0.3 (SD 1.1) with 21 (75%) of the children a normal weight for their age, 5 (17.9%) overweight and 2 (7.9%) obese.

**Table 2:** Cohort BoNTA treatment details

Study ID number/ Age Group yrs. at assessment	CP type Predominate tone	Comorbidity BMI	Current GMFCS/MACS/CFCS	Details of BoNTA treatments						Lower limb surgery	GMFM
				Total #LL	Age (yrs) first dose	Age (yrs) last dose	BoNTA dose u/kg Mean (SD)	BoNTA u/kg/muscle Mean (SD)	Upper limb treatment	Total surgeries/ Age first surgery/type No. BoNTA doses pre-surgery/post-surgery	Total score (SEM) 95% CI/Centile
#1/6–8	Hemiplegia Dystonia	No 19.1	II/I/I	4	6	8	6.7 (0)	1.9 (0.3)	Yes	0	70.8 (1.6) 67.7–73.9/65th
#2/6–8	Hemiplegia^a^ Spasticity	Autism 16.1	II/III/I	7	5	9	4.6 (1.1)	2.6 (0.3)	Yes	0	70.0 (1.5) 67–73/60th
#3/6–8	Hemiplegia Spasticity	CHD 16.7	II/II/I	5	3	6	8.6 (2.6)	4.5 (0.9)	Yes	0	83.0 (2.6) 77.9–88/95th
#4/9–10	Hemiplegia Spasticity	CHD 17.0	I/II/I	8	4	8	4.7 (0.9)	2.2 (0.7)	No	0	88 (3.4) 81.3–94.7/50th
#5/9–10	Hemiplegia^a^ Spasticity	No 15.1	I/II/I	11	3	8	5.3 (0.6)	5.3 (0.6)	No	0	80.9 (2.5) 76.1–85.8/25th
#6/9–10	Hemiplegia Dystonia	No 15.7	II/I/I	4	5	9	6.1 (1.2)	3.1 (0.6)	No	0	76.0 (1.9) 72.2–79.8/50th
#7/11–12	Hemiplegia Spasticity	Autism 29.8	II/V/I	8	4	9	4.1 (1.5)	2.6 (0.4)	Yes	0	51.6 (1.2) 49.1–54.0/< 3rd
#8/11–12	Hemiplegia Spasticity	Epilepsy 20.6	II/I/I	11	3	10	3.2 (1.1)	2.3 (0.5)	Yes	0	72.6 (1.6) 69.4–75.8)/55th
#9/11–12	Hemiplegia^a^ Spasticity	No 18.2	I/II/I	18	2	10	4.4 (2.0)	4.4 (2.0)	Yes	0	81.9 (2.3) 77.3–86.5/25th
#10/11–12	Hemiplegia Dystonia	Epilepsy, ID 16.0	I/II/I	9	5	11	4.8 (1.0)	2.4 (0.5)	No	0	89.7 (4.1) 81.6–97.8/55th
#11/13–15	Hemiplegia^a^ Spasticity	Autism, ID 20.9	II/IV/IV	21	3	15	6.6 (2.5)	4.0 (1.8)	Yes	1/11 yrs. /Gait Pre 15/Post 6	64.0 (1.4) 61.2–66.7/20th
#12/16 and over	Hemiplegia Dystonia	No 18	I/I/I	13	10	15	2.1 (1.0)	2.1 (1.0)	Yes	0	96.0 (8.2) 79.0–100/85th
#13/16 and over	Hemiplegia Spasticity	No 19.1	II/I/I	19	1	16	7.3 (3.5)	3.2 (1.4)	Yes	1/9 yrs./Gait Pre 14/Post 5	75.3 (1.9) 71.7–79.0/ 65th
#14/6–8	Diplegia^a^ Spasticity	No 16.6	II/I/I	12	3	7	4.6 (1.1)	2.6 (0.3)	Yes	0	74.7 (1.9) 70.9–78.6/85th
#15/6–8	Diplegia Spasticity	No 15.2	II/I/I	11	1	8	16 (4.2)	4.3 (0.6)	No	0	78.3 (2.1) 74.1–82.4/90th
#16/9–10	Diplegia Spasticity	LD 23	II/I/I	13	4	10	9.3 (2.1)	3.2 (0.7)	No	0	68.9 (1.5) 66.0–71.7/45th
#17/9–10	Diplegia Spasticity	No 19.2	I/I/II	0	NA	NA	NA	NA	No	0	85.2 (2.8) 79.7–90.8/35th
#18/9–10	Diplegia Spasticity	No 13.4	II/I/I	11	2	10	9.3 (3.2)	4.1 (0.8)	No	0	72.6 (1.6)) 69.4–79.8/60th
#19/9–10	Diplegia Spasticity	Autism 18.7	II/III/I	21	2	10	15.2 (2.2)	3.4 (0.7)	No	0	67.4 (1.5) 64.5–70.3/35th
#20/9–10	Diplegia^a^ Spasticity	No 20.4	II/I/I	15	1	7	11.6 (2.1)	2.8 (0.6)	Yes	1/9 yrs./Gait Pre 15 Post 0	68.9 (1.5) 66.0–71.7/40th
#21/11–12	Diplegia Spasticity	ID, ILD 15	II/III/III	1	10	10	7.7 (NA)	3.9 (NA)	No	0	72.6 (1.6) 69.4–75.8/55th
#22/11–12	Diplegia Spasticity	ID 18.1	II/I/II	7	8	11	10.0 (1.2)	1.8 (0.2)	No	0	78.3 (2.2) 73.9–82.7/75th
#23/11–12	Diplegia^a^ Spasticity	PNI, LD 21	II/II/I	18	2	11	7.8 (4.9)	3.3 (1.0)	Yes	1/7 yrs./Gait Pre 11 Post 7	61.5 (1.3) 59.0–64.0/10th
#24/11–12	Diplegia ^a^ Spasticity	Epilepsy 24.4	II/II/II	17	2	10	13.3 (3.2)	3.5 (1.4)	No	0	70.0 (1.5) 67.0–73.0/40th
#25/11–12	Diplegia ^a^ Spasticity	CHD 23	II/I/I	14	6	12	5.6 (2.3)	1.7 (0.6)	No	0	76.0 (2.1) 72.0–80.0/65th
#26/11–12	Diplegia ^a^ Spasticity	No 28.6	II/I/I	2	7	9	1.4 (0.3)	1.4 (0.3)	No	0	74.2 (1.8) 70.7–76.7/60th
#27/13–15	Diplegia Dystonia	No 20.2	II/III/II	27	2	16	9.0 (2.1)	2.8 (1.2)	No	0	86.5 (3.10 80.5–92.5/90th
#28/11–12	Diplegia	No 23.2	II/I/I	6	7	10	5.6 (2.3)	1.7 (0.6)	No	0	83.0 (2.5) 78.1–87.8/85th

*MACS* Manual Ability Classification System, *CFCS* Communication Function Classification System, *CHD* congenital heart disease, *ID* intellectual disability, *LD* learning difficulties, *ILD* interstitial lung disease, *PNI* peripheral nerve injury, *NA* not applicable

^a^Pain reported present on brief pain inventory. Age at assessment listed as range to maintain deidentified status

**Fig. 2:**
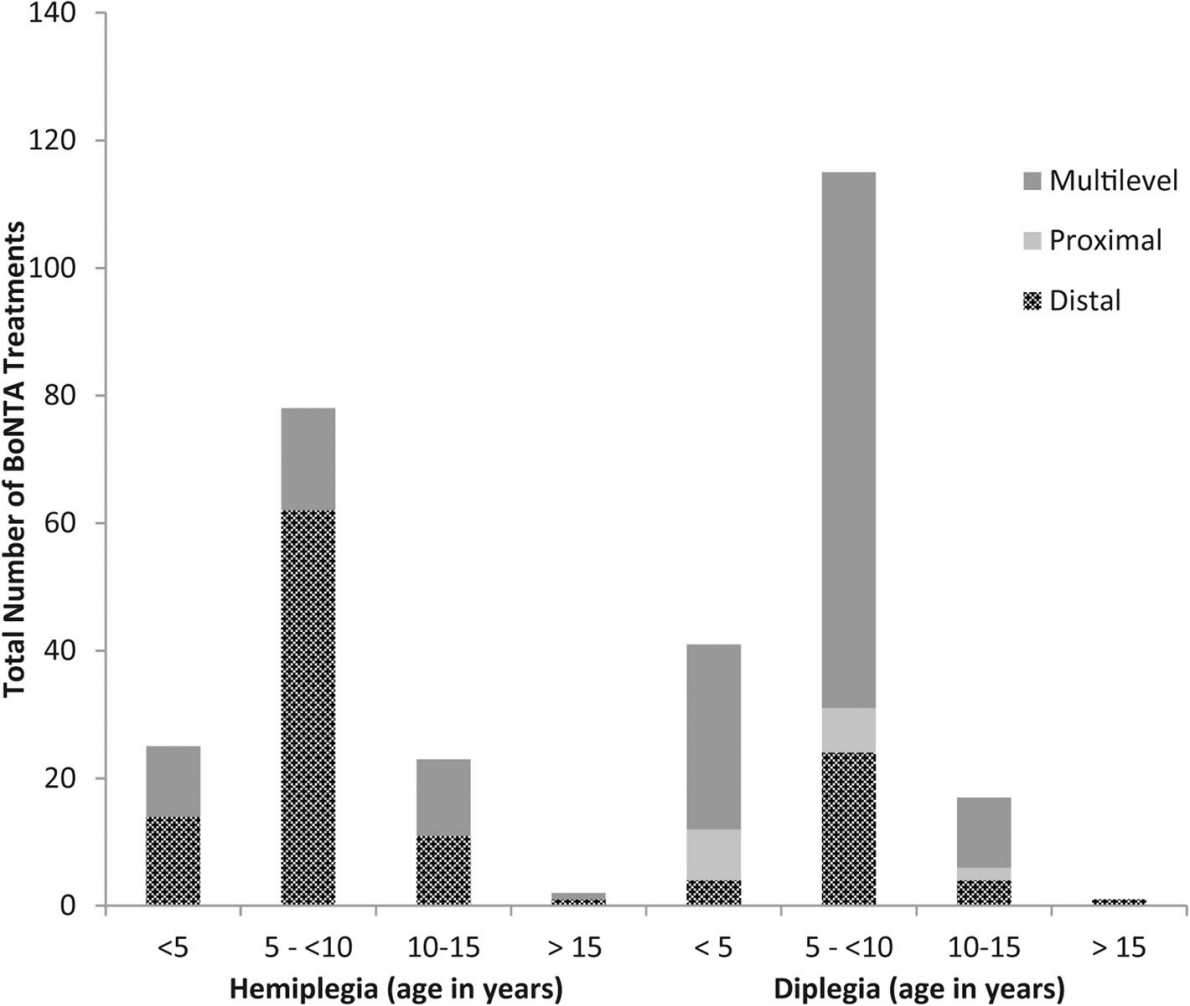
Total number of BoNTA treatment by CP topography and age

Of the 28 children in this cohort, 27 received treatment with BoNTA, the median (IQR) total dose of BoNTA to lower limbs per treatment was 6.95 u/kg (4.5, 11) and the median (IQR) dose of BoNTA u/kg/muscle was 2.95 (2.2, 4). The distribution of the BoNTA in the lower limb muscles by age and topography is documented in Fig. 2. There was a higher use of BoNTA in multilevel muscles in children with diplegia compared to those with hemiplegia (*p* < 0.001). None of the 28 children were on any additional medication to modulate tone or movement disorders. For children with hemiplegia the average time between injections was 8.5 months (SD 2.4 months) and for children with diplegia the average time between injections was 7.2 months (SD 3.6 months).

**Fig. 3:**
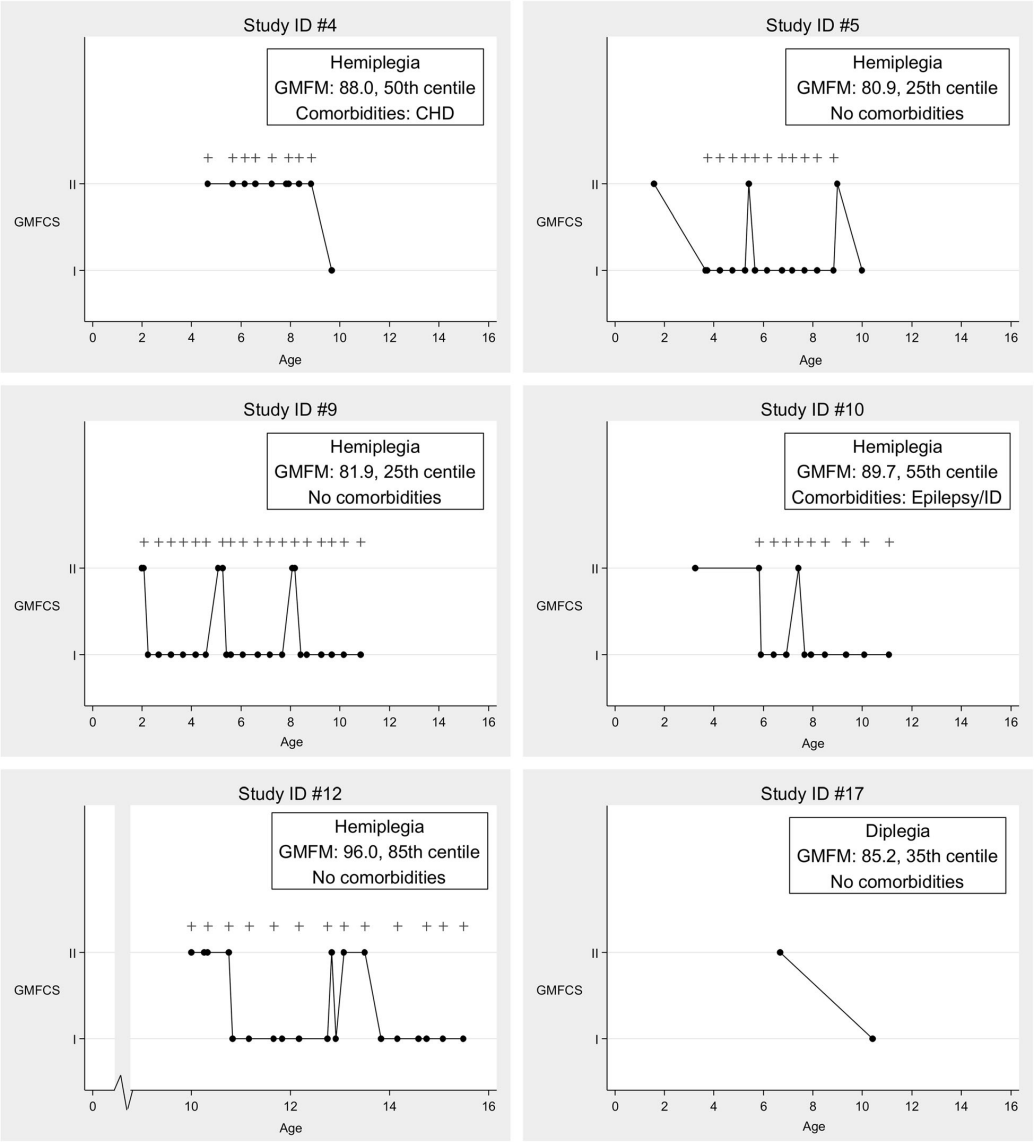
Treatment details and GMFM results six individuals who improved from GMFCS II to GMFCS I, + represent BoNTA treatments

For all 28 subjects there was a median (IQR) of 15 (8.5, 22) GMFCS recordings done. Of these 28 children, six (21.4%) improved to GMFCS level I. The average GMFM-66 centile score for age and GMFCS level for these six children was 46, with a mean GMFM-66 score of 86.9. The age when BoNTA treatments were received, the recorded GMFCS and GMFM level, and the comorbidities of these six children are documented in Fig. 3. Notably, five of these six individuals had a hemiplegic distribution and only two of the six had comorbidities.

Of the 22 children who remained GMFCS level II at time point 2, the average GMFM-66 centile for GMFCS level II was 56.7, with a mean GMFM-66 score of 72.55 and average age of 11.2 years. Only eight of the 22 children received the same GMFCS rating at all assessments, with a median (IQR) of 16 (7, 19) GMFCS recordings. No child increased their final GMFCS level recording, that is, deteriorated in gross motor function.

The Brief Pain Inventory – Short Form was completed by 26 of the 28 enrolled children. Pain, other than everyday kinds of pain such as toothache or minor headache, was present in 10 of the 28 children (38.5%), and the average pain rating was 3 out of 10 (SD 2.4). Of those 10 children with pain, 5 (50%) felt the pain interfered with their general activity and rated the average amount of interference as median (IQR) of 1(0,7). Of the 10 children with pain, 5 (50%) had comorbidities; and of the 18 children with no pain, 10 (56%) had comorbidities, a similar ratio. There was no association with pain being present and a BMI in the obese or overweight range (*p* = 0.157).

The median (IQR) PEM-CY scores for all of the cohort participation in the three domains were: home 6.1 (5.4,6.5), school 3.6 (3,4,4.6) and community 2.2 (1.6,3.0). There was no statistically significant relationship between the PEM-CY score and topography, final GMFCS level or pain scores, see Table 3. Correlation and linear regression of PEM-CY and GMFM is shown in Table 4, with no significant association between these two measures in either home or community. Interestingly there was a statistically significant negative association between school participation and GMFM centile with a correlation coefficient of − 0.5 (*p* = 0.010).

**Table 3:** Participation and Environment Measure for Children and Youth (PEM-CY) and the relationship with topography, GMFCS and pain

	n (%)	Participation and Environment Measure for Children and Youth (PEM-CY)					
		Home Average		School Average		Community Average	
		median (IQR)	*p* value	median (IQR)	*p* value	median (IQR)	*p* value
Total PEMCY Complete	25 (89%)	6.1 (5.4, 6.5)	NA	3.6 (3.4, 4.6)	NA	2.2 (1.6, 3.0)	NA
Topography							
Diplegia	14 (56.0%)	6.1 (5.2, 6.5)	0.826	3.5 (3.0, 5.0)	0.659	2.1 (1.6, 3.9)	0.510
Hemiplegia	10 (40.0%)	6.2 (5.4, 6.5)		3.8 (3.4, 4.4)		2.5 (2.1, 3.0)	
GMFCS							
I	5 (20.0%)	6.2 (5.4, 6.5)	0.864	3.4 (3.4, 4.4)	0.784	2.8 (1.4, 3.0)	0.634
II	20 (80.0%)	6.1 (5.3, 6.5)		3.7 (3.2, 4.6)		2.2 (1.8, 3.5)	
Pain Present							
0(NA)	2 (8.0%)	6.4 (6.4, 6.4)	0.397	3.7 (3.4, 4.0)	0.953	6.0 (5.2, 6.5)	0.398
1 (Yes)	8 (32.0%)	6.2 (6.0, 6.6)		3.6 (3.1, 5.1)		3.6 (3.4, 4.6)	
2 (No)	15 (60.0%)	6.0 (5.2, 6.5)		3.6 (3.4, 4.6)		2.2 (1.6, 2.8)	
BMI							
normal weight	19 (76.0%)	6.2 (5.4, 6.5)	0.659	3.6 (3.4, 4.6)	0.903	2.2 (1.6, 3.0)	0.781
overweight	4 (16.0%)	6.0 (5.2, 6.2)		3.7 (3.2, 5.3)		2.3 (1.4, 3.8)	
obese	2 (8.0%)	5.9 (5.0, 6.7)		3.7 (3.2, 4.2)		3.2 (2.1, 4.4)

**Table 4:** Participation and Environment Measure for Children and Youth (PEM-CY) and Gross Motor Function Measure 66 (GMFM-66) association. GMFCS II only (*n* = 20)

	GMFM Total Score		
	coefficient (95% CI)	*p* value	correlation coefficient (95% CI)
PEMCY (outcome)			
Home average	0.04 (−0.02, 0.09)	*p* = 0.146	0.34 (−0.12 to 0.68)
School average	−0.05 (−0.11, 0.02)	*p* = 0.143	−0.34 (−0.68 to 0.12)
Community average	−0.02 (− 0.09, 0.05)	*p* = 0.574	− 0.13 (− 0.54 to 0.33)
GMFM centile (predictor)			
Home average	0.01 (− 0.01, 0.03)	*p* = 0.219	0.29 (− 0.18 to 0.65)
School average	−0.02 (− 0.04, − 0.01)	*p* = 0.014	−0.54 (− 0.79 to − 0.13)
Community average	−0.01 (− 0.04, 0.01)	*p* = 0.167	−0.32 (− 0.67 to 0.14)

## Discussion

Our study confirms that the majority of ambulant children treated at a young age with repeated doses of BoNTA within an integrated comprehensive service, maintain their functional motor levels over time, as documented by the GMFCS level. The rates of pain and participation in our cohort are similar to that documented in other populations [35–39]. Our rates of GMFCS stability in this treated population are also similar to those recently documented in large cohort studies [40, 41]. As the GMFCS is a classification system rather than an outcome measure, we used the GMFM − 66 [30] to look at our population’s motor function in more detail. As documented by Hanna et al. for children in GMFCS levels I and II, the average age at which children achieve 90% of their expected limit in GMFM-66 motor ability is 5 years 2 months for GMFCS I and 4 years 11 months for GMFCS II, they found no evidence of functional decline, on average, for children in GMFCS levels I and II [42]. The median age of our cohort at the time of GMFM-66 assessment was 11.5 years, so it can be presumed that at the age at which we assessed GMFM-66 our patients have achieved motor stability. Importantly we have shown that in this highly treated population, the average GMFM-66 limit of our children in GMFCS level II is 72.55, which is consistent with the mean of 68.5 reported by Hanna [42]. For our children who became GMFCS level I, the average GMFM-66 limit was 86.9, again consistent with the average of 89.5 reported by Hanna et al. [42] Figure 3 provided detailed information on the small case series of patients who improved GMFCS level following treatment in our comprehensive service. For patients 12 and 4, both of whom have received multiple series of BoNTA treatments, we propose that these patients have permanently changed GMFCS level as they had their first recordings of GMFCS level II made after the age of 4 years, which is when the GMFCS level is considered stable, and their GMFM-66 scores are in the high centile range, being at the 85th and 50th centile respectively for GMFCS level.

In the original GMFCS motor curves, children who had received BoNTA or intrathecal baclofen or who had undergone selective dorsal root rhizotomy were excluded as it was not then known how these relatively new interventions would influence gross motor function [43]. This study details the BoNTA interventions provided and confirms that the majority of our highly treated population remains at a stable GMFCS level and with the GMFM-66 average consistent with the original published average levels. Notably, in a significant percentage of our assessed population, the GMFCS improved over time and deteriorated in none. The decision to use BoNTA is multifactorial and guided by the CPMS model of goal-based decision making within the International Classification of Functioning, Disability and Health (ICF) model with input from a multidisciplinary team, parent(s) and, where appropriate, the child. In our clinical service the outcome of BoNTA treatment is evaluated by review of the child post BoNTA treatment that includes history for side effects, documentation of a technical response e.g. by change in Modified Ashworth, Modified Tardieu assessments, or reduction in spasm scores or Barry Albright dystonia score, and documentation of outcome of goals. A written report is submitted by the community therapists of the post intervention provided and information on the outcome of goals is also commented on in this report. Our doses of BoNTA are low to moderate [2] and our distribution of muscle use is similar to that of other Australian teams. All patients who receive BoNTA in our service must have a community therapy provider and when our patients receive medical or surgical intervention through our program, we also provide funding for extra post-intervention therapy sessions from the community team. For example, if a patient receives lower limb BoNTA to one or to two limbs, they receive eight or 16 extra therapy sessions respectively.

It is well understood that comorbidities in children with CP affect the outcome, our rates of comorbidities are similar to that documented by Novak [44] and the Australian Cerebral Palsy Register [45], and as expected the rate of comorbidities in those children who improved GMFCS level (33%) was lower than in those whose GMFCS level remained stable (54%). Increasing BMI is a significant issue for children of all abilities but a significant further risk factor for children with a motor impairment. Our rates of overweight and obese children are similar to those seen in typically developing children and children with CP [46–48].

The dimension of participation is an important inclusion in the ICF [49] and as it is clear that participation contributes to quality of life [50] an important target of our treatment is to provide increased participation. In our cohort of children there was no statistically significant relationship between PEM-CY scores and topography, final GMFCS level or pain scores. The statistically significant negative association between school participation and GMFM is not easily understood and likely multifactorial. Anecdotally what is seen is that children with CP who have good motor function but not the level of motor function equivalent to that of typically developing children are or tend to be isolated in motor-based school activities as they cannot keep up with their peers.

It is known from recent large population cohort studies on GMFCS level stability that a percentage of patients in each GMFCS level change levels over time and there have been recent calls in the literature to study the comorbidities and treatments received by those subgroups of children with a permanent change in GMFCS level [40, 51]. To our knowledge, this study is the first to provide detailed information on medical interventions and comorbidities of individuals with CP in relationship to GMFCS level stability.

### Limitations

The major limitation of this study is the absence of a GMFM-66 assessment at time point 1. As the aim of this study was to look at GMFCS stability in a treated population we limited our cohort to children whose first GMFCS level recorded was level II as these children have potential to change GMFCS level in both directions, but motor function is not reported to decline in adolescence [42]. Only a randomly selected 28 of the 40 eligible children were assessed. Table 1 suggests that they may have been a relatively good outcome group, but the differences are not marked. This study focuses on BoNTA treatment as this is the most frequent major intervention at this GMFCS level, in particular this study does not provide details concerning the type of surgery. We do not report any adverse side effects of BoNTA in this paper since there have been several recent papers on this subject [9, 10, 52] including our own [53]. The impact of repeat dosing with BoNTA on muscle structure and function has not been studied in this paper but is recognised to be an important consideration in the long-term use of BoNTA. We have recently published on the impact on muscle volume and muscle structure following repeat dosing of BoNTA and presently we aim to minimise the dose of BoNTA used, rotate muscle selection where possible and ensure post intervention strength training, where appropriate [54–56]. It is now recognised that children with CP from socio-economically disadvantaged settings are more likely to have reduced motor functional outcomes [57]; this study has not looked at any socio-economic determinants of health but this would be important in future research.

## Conclusion

This cohort study has confirmed that children with CP and a GMFCS level of II treated at a young age with repeated low to moderate doses of BoNTA within an integrated comprehensive service, maintain their functional motor gains at a later age.

## Data Availability

The datasets generated and/or analysed during the current study are not publicly available due to the guidelines of our Ethics Committee process. Data and material will be available upon reasonable request as deidentified data and after review of request by our institution’s ethics committee, applications should be directed to the corresponding author at, jane.valentine@health.wa.gov.au.

## Abbreviations

BMI: Body mass index
BoNTA: Botulinum toxin type A
CFCS: Communication Function Classification System
CHD: Congenital heart disease
CP: Cerebral Palsy
CPMS: Cerebral Palsy Mobility Service
GMAE-2: Gross Motor Ability Estimator
GMFCS: Gross Motor Function Classification System
GMFM-66: Gross Motor Function Measure
ICF: International Classification of Functioning, Disability and Health
ID: Intellectual disability
ILD: Interstitial lung disease
IQR: Interquartile range
LD: Learning difficulties
MACS: Manual Ability Classification System
NA: Not applicable
PCH: Perth Children’s Hospital
PEM-CY: The Participation and Environment Measure for Children and Youth
PNI: Peripheral nerve injury
SD: Standard Deviation
WA: Western Australia
